# Impact of childhood trauma on impulsivity in patients with bipolar disorder

**DOI:** 10.1192/j.eurpsy.2022.433

**Published:** 2022-09-01

**Authors:** D. Bougacha, S. Ellouze, R. Jenhani, R. Ghachem

**Affiliations:** 1 Razi hospital, B, Manouba, Tunisia; 2 Hedi Chaker University Hospital, Psychiatry, Sfax, Tunisia

**Keywords:** bipolar disorder, Childhood Trauma, Impulsivity

## Abstract

**Introduction:**

Childhood trauma has been demonstrated to be associated with several indicators of worse course in bipolar disorder (BD). Links between early adversity and the complexity of the disorder might be mediated by various dimensions of psychopathology, such as impulsivity.

**Objectives:**

The aim of this study was to investigate the impact of traumatic childhood experiences on impulsivity in individuals with bipolar disorder.

**Methods:**

We conducted a cross-sectional, descriptive, and analytical study. Sixty-one euthymic patients with bipolar disorder were recruited in the department of psychiatry B of Razi Hospital, during their follow-up. The Childhood Trauma Questionnaire (CTQ) and the Barratt Impulsiveness Scale-11 (BIS-11) were used to assess childhood traumatic experiences and impulsivity.

**Results:**

The mean age of patients was 43.4. The sex ratio was 2.4. The mean score obtained on the Bis-11 scale was 74.8. More than half of patients (53%) had high levels of impulsivity. Almost two-thirds of patients (64%) had experienced at least one type of childhood trauma. Higher scores on the various dimensions of childhood trauma apart from physical neglect, were significantly associated with higher total BIS-11 score as well as with all its subscales. Linear regression with the CTQ total score as the independent variable showed a statistically significant effect of childhood trauma score on attentional impulsivity.

**Conclusions:**

Our findings suggest that interventions that target impulsive behavior in individuals with bipolar disorder should pay particular attention to traumatic childhood experiences. Furthermore, early identification and management of childhood trauma may reduce levels of impulsivity and thus improve the outcome and prognosis of bipolar disorder.

**Disclosure:**

No significant relationships.

